# Supplementary material for: TbpBY167A-Based Vaccine Can Protect Pigs against Glässer’s Disease Triggered by Glaesserella parasuis SV7 Expressing TbpB Cluster I
Source: Pathogens. 2022 Jul 4;11(7):766. doi: 10.3390/pathogens11070766 (PMC9323293; doi:10.3390/pathogens11070766)
Supplement: Supplementary file 1 [file pathogens-11-00766-s001.zip › pathogens-1758272-supplementary.pdf]

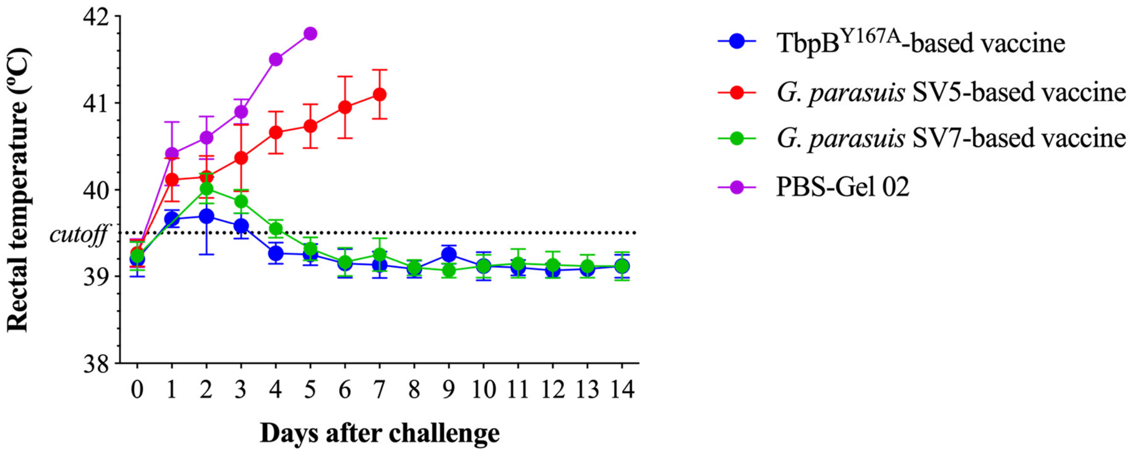

Figure S1. Rectal temperature recording of the pigs after challenge.

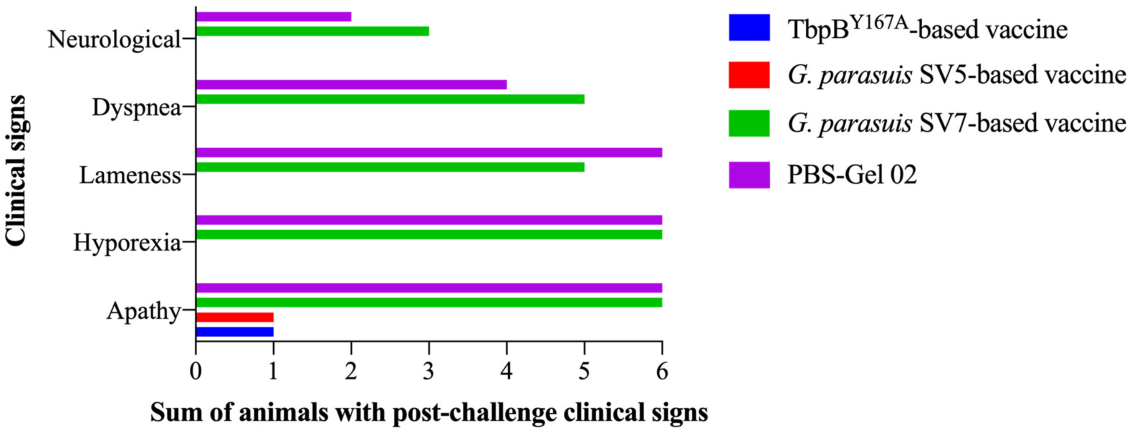

Figure S2. Clinical signs recording of the pigs after challenge. On the X axis is represented the cumulative number of animals per group that showed a certain clinical sign over the post-challenge period.
